# The immune microenvironment landscape shows treatment-specific differences in rectal cancer patients

**DOI:** 10.3389/fimmu.2022.1011498

**Published:** 2022-09-27

**Authors:** Cristina Graham Martínez, Yari Barella, Sonay Kus Öztürk, Marleen Ansems, Mark A.J Gorris, Shannon van Vliet, Corrie A.M Marijnen, Iris D Nagtegaal

**Affiliations:** ^1^ Department of Pathology, Radboud University Medical Centre, Nijmegen, Netherlands; ^2^ Radiotherapy & OncoImmunology Laboratory, Department of Radiation Oncology, Radboud University Medical Centre, Nijmegen, Netherlands; ^3^ Department of Tumor Immunology, Radboud University Medical Centre, Nijmegen, Netherlands; ^4^ Oncode Institute, Utrecht, Netherlands; ^5^ Department of Radiotherapy, Netherlands Cancer Institute, Amsterdam, Netherlands; ^6^ Department of Medical Oncology, Leiden University Medical Centre, Leiden, Netherlands

**Keywords:** immune microenvironment, response, rectal cancer, neoadjuvant treatment, immune landscape

## Abstract

Neoadjuvant therapy is the cornerstone of modern rectal cancer treatment. Insights into the biology of tumor responses are essential for the successful implementation of organ-preserving strategies, as different treatments may lead to specific tumor responses. In this study, we aim to explore treatment-specific responses of the tumor microenvironment. Patients with locally advanced adenocarcinoma of the rectum who had received neo-adjuvant chemotherapy (CT), neo-adjuvant radiochemotherapy (RCT), neo-adjuvant radiotherapy with a long-interval (LRT) or short-interval (SRT) or no neoadjuvant therapy (NT) as control were included. Multiplex-immunofluorescence was performed to determine the presence of cytotoxic T-cells (T-cyt; CD3+CD8+), regulatory T-cells (T-reg; CD3+FOXP3+), T-helper cells (T-helper; CD3+CD8-FOXP3-), B cells (CD20+), dendritic cells (CD11c+) and tumor cells (panCK+). A total of 80 rectal cancer patients were included. Treatment groups were matched for gender, tumor location, response to therapy, and TNM stage. The pattern of response (shrinkage *vs.* fragmentation) was, however, different between treatment groups. Our analyses reveal that RCT-treated patients exhibited lower stromal T-helper, T-reg, and T-cyt cells compared to other treatment regimens. In conclusion, we demonstrated treatment-specific differences in the immune microenvironment landscape of rectal cancer patients. Understanding the underlying mechanisms of this landscape after a specific therapy will benefit future treatment decisions.

## 1 Introduction

In recent times, the prognosis of patients with rectal cancer (RC) has improved immensely (1). Neoadjuvant therapy is the cornerstone of RC treatment, allowing for easier resections and better clinical outcomes (2, 3). The presence of various degrees of tumor response demonstrates organ-preserving treatments are possible in selected patients (4, 5). To fully exploit the organ-preserving potential, we need to fully understand this response and the role of the tumor microenvironment in this process.

Different types of treatments have different clinical effects. A simple comparison of pathological complete response (pCR) rates already illustrates this. Neoadjuvant radiochemotherapy (RCT) has increased pCR rates, with an incidence of approximately 14% of patients (6). Lower pCR rates are present in patients treated with 5x5Gy radiotherapy (RT), depending on the treatment interval; either short (SRT) (reported pCR of 0.3% but increasing after 4 week waiting period) or long wait (LRT) (pCR range 9.3%-10.4%) (7, 8). New clinical trials with different combinations of chemotherapy and, especially, wait intervals provide promising results, in some cases reaching 28% pCR rates (6). In general, longer waiting time after initial treatment and more intense local therapy are considered to improve pCR and several oncological outcomes (9).

Previous studies have shown the potential role of immune infiltrates in the prediction of radio-responsiveness to neoadjuvant RCT in rectal cancer (10). These improved outcomes might also be induced by changes in the immune cell and cytokine composition as a response to therapy, and may, therefore, be therapy-specific. The immune microenvironment of RC is complex, different from colon cancer, with variable prognostic impact of individual types of immune cells, such as tumor-infiltrating T-regs (11–17). The well-established role of CD3+ and CD8+ cells in colorectal cancer (i.e., the immunoscore (18)) has paved the road to investigate the impact of other immune cell subsets. CD11c+ (dendritic cells (DCs)) (19, 20), CD20+ (B cells) (21, 22), CD3+CD8-FOXP3-(T-helper cells) (10), CD3+FOXP3+[T-regulatory cells (T-regs)]] (23, 24) and CD3+CD8+[cytotoxic T cells (T-cyt)] cells (25, 26) have been some of the major targets of immune-profiling in recent years. In newly diagnosed rectal cancers, several studies have elegantly shown an increased presence of T-helper and cytotoxic T cells prior to RCT correlates with better response (27, 28) and better recurrence-free survival (29).

Since local treatment interferes with this microenvironment, investigating the repopulation of the immune infiltrate after each type of therapy is crucial to understanding the biology of the tumor response. Some studies have shown differences in the circulating subpopulations of immune cells throughout therapy or after it (30, 31), but, to the best of our knowledge, little is known about specific changes in the tumor and tumor microenvironment locally, in relation to tumor response. We have recently described a biology-based classification of tumor response (32) that allows us to integrate the tumor response and the microenvironment. According to this, a partial response can be classified into a fragmented (disintegration of the tumor mass in different sized and shaped fragments) or shrinkage (downsizing of tumor mass) pattern of response which has prognostic implications for the patient. Our integrative approach allows us to compare the effects of specific therapeutic strategies in order to elucidate relevant differences in the microenvironment.

Here, we explore the effects and interactions of the different types of treatment on tumor cells and microenvironment and correlate this with a biologically meaningful and clinically relevant response scoring method.

## 2 Methods

### 2.1 Patient cohort and material used

From an original in-house cohort of 728 rectal cancer (RC) patients from the Radboud University Medical Center Nijmegen, a total of 80 patients with adenocarcinoma NOS (not otherwise specified) of the rectum were selected to be included in this study. Patients with known hereditary colorectal cancer were excluded. An opt-out system for ethical approval was in place. Patient material was obtained from the pathology archives of the Radboud University Medical Centre, Nijmegen, The Netherlands. Selected patients received one of four different treatment regimens between 2015 and 2019; neo-adjuvant chemotherapy (CT), neo-adjuvant radiochemotherapy (RCT), neo-adjuvant radiotherapy with a long interval (LRT), or neo-adjuvant radiotherapy with a short interval (SRT), with no neo-adjuvant therapy (NT) serving as control. Per group, 16 patients matched (as much as possible) for gender, tumor location, and cTNM stage were included (details are shown in **Table 1**).

**Table 1:** Relevant patient information.

Variable	NT n(%)	CT n(%)	RCT n(%)	LRT n(%)	SRT n(%)	P
**Age, median (IQR)**	65 (39-84)	60 (29-78)	64 (46-80)	67 (49-79)	74 (40-87)	0.02
**Gender**						0.44
Male	9 (56%)	9 (56%)	8 (50%)	12 (75%)	12 (75%)	
Female	7 (44%)	7 (44%)	8 (50%)	4 (25%)	4 (25%)	
**Tumor location**						0.02
Rectum	7 (47%)	12 (75%)	14 (88%)	15 (94%)	14 (88%)	
Rectosigmoid	7 (47%)	4 (25%)	0 (0%)	0 (0%)	2 (12%)	
LAR (low anterior res)	1 (6%)	0 (0%)	2 (12%)	1 (6%)	0 (0%)	
**Differentiation grade**						0.17
Good	16 (100%)	16 (100%)	15 (94%)	15 (94%)	13 (81%)	
Poor	0 (0%)	0 (0%)	1(6%)	1(6%)	3 (19%)	
**(c)LN involvement**	10 (100%)	14 (100%)	14 (100%)	13 (100%)	13 (100%)	<0.001
Yes	2 (20%)	13 (93%)	13 (93%)	11 (85%)	10 (77%)	
No	8 (80%)	1 (7%)	1 (7%)	2 (15%)	3 (23%)	
**Angioinvasion**						0.13
Yes	10 (62%)	6 (38%)	6 (38%)	7 (44%)	12 (75%)	
No	6 (38%)	10 (62%)	10 (62%)	9 (56%)	4 (25%)	
(y*)pT category						<0.001
1	0 (0%)	0 (0%)	0 (0%)	0 (0%)	2 (13%)	
2	8 (50%)	3 (18%)	7 (44%)	5 (31%)	1 (6%)	
3	7 (44%)	7 (44%)	9 (56%)	10 (63%)	13 (81%)	
4	1 (6%)	6 (38%)	0 (0%)	1 (6%)	0 (0%)	
(y*)pN category						0.07
0	14 (88%)	4 (25%)	10 (63%)	7 (44%)	9 (57%)	
1	1 (6%)	7 (44%)	4 (25%)	6 (38%)	5 (31%)	
2	1 (6%)	5 (31%)	2 (12%)	3 (18%)	2 (12%)	
(y*)pM category						0.11
0	15 (94%)	12 (75%)	16 (100%)	16 (100%)	16 (100%)	
1	1 (6%)	4 (25%)	0 (0%)	0 (0%)	0 (0%)	
**Downstaging**						0.97
Progression	*	1 (6%)	0 (0%)	1 (6%)	1 (6%)	
No change	*	6 (38%)	6 (38%)	7 (44%)	6 (38%)	
Downstage	*	7 (44%)	9 (56%)	6 (38%)	6 (38%)	
Unknown	*	2 (12%)	1 (6%)	2 (12%)	3 (18%)	
**Regression**						0.04
Partial	*	13 (81%)	16 (100%)	12 (75%)	16 (100%)	
No	*	3 (19%)	0 (0%)	4 (25%)	0 (0%)	
**Pattern of response**						0.02
Shrinkage	8 (62%)*	0 (0%)	6 (37%)	7 (64%)	7 (50%)	
Fragmentation**	5 (38%)*	12 (100%)	10 (63%)	4 (36%)	7 (50%)	

This table shows the clinical variables of our cohort. Distribution of patients per treatment group with data as n/(%).

*Cannot be assessed because no therapy was given. **14 cases with No response were excluded.

**Figure 1:**
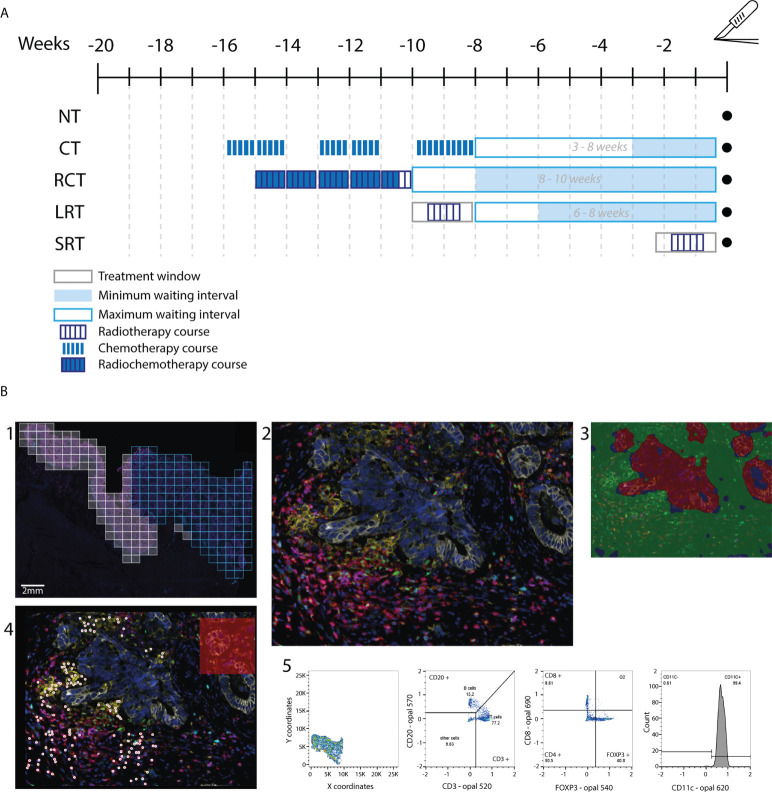
Flow diagram of the study. **(A)** Treatment schedule for included patients. Patients received one in 4 neoadjuvant therapy regimens. Treatment window, waiting period between therapy and surgery, and dose fractions are indicated. **(B)** Workflow followed for the immunophenotype quantification. In 1, the whole slide image is selected and regions of interest (ROIs) are drawn to scan at 20x. In blue we see an example of included ROIs, as the white ROIs on the left of the image are excluded as they contain normal rectal epithelium. 2, An example of a ROI output. 3, Tissue segmentation training leads to a quite accurate region classification. Red=tumor, green=stroma, blue=background. 4, Example of the cell phenotyping output from the in-house AI pipeline used. 5, Example of the gating used to discern immune cell populations. NT, No therapy; CT, Chemotherapy; RCT, Radiochemotherapy; LRT, Radiotherapy long course; SRT, Radiotherapy short course; BT, brachytherapy.

For each patient, two consecutive slides were cut from the most representative block of the primary tumor and were stained for H&E and an immunofluorescence (IF)-multiplex panel (33) (**Figure 1**).

### 2.2 Mutation analysis and MSI status

Sufficient material was present for 64 out of the 80 patients for molecular analysis. A targeted mutational panel (PATHv3) used in routine diagnostics was used to determine any mutations that could clearly influence the immune phenotype of patients (exact panel targets can be found in **Supplementary Table 2**).

### 2.3 Immunoflourescence stains

The multiplex IF staining protocol Opal™ 7 Tumor Infiltrating Lymphocyte (TIL) Kit (OP7TL3001KT, Akoya Biosciences, MA, USA), was modified and optimized for rectal 4µm thick FFPE tissue sections (34). In short, slides were sequentially stained using antibodies against CD8, CD20, CD3, Foxp3, CD11c, and pan-Cytokeratin AE 1/3 using the BOND RX automated research stainer (Leica Biosystems, Wetzlar, Germany). The anti-CD45RO used in a previous study (35) was exchanged for CD11c (ab52632, Abcam, Cambridge, UK, RRID: AB_2129793). Concentrations of antibodies, retrieval steps, and corresponding Opal dyes were adjusted (details available in **Supplementary Table 1**) to ensure the best signal intensity for each marker that would allow exposure times of 15-100 ms on multispectral regions. Slides were manually stained with DAPI and mounted using Fluoromount-G® (0100-01, Southern Biotech, ALA, USA, RRID: SCR_015961).

### 2.4 Immunofluorescence imaging and data processing

Imaging was performed using the VECTRA 3 Quantitative Pathology Imaging System (PerkinElmer) and Vectra V3.0.4 software (PerkinElmer®, Hopkinton, MA, USA). All standard epi-fluorescent filters were used; DAPI, FITC, CY3, Texas Red, and CY5. Whole slide scans were acquired using x4 magnification and subsequently scanned at x20 magnification for the multispectral regions of interest (**Figure 1B**). Images were first processed using the inForm software (V.2.4.8, Akoya Biosciences, MA, USA, RRID: SCR_019155) for cell and tissue segmentation and then processed through an in-house AI pipeline to phenotype immune cells, which were thereafter analyzed in FlowJo™ (Ashland, OR, USA, RRID: SCR_008520) as previously described (35, 36).

### 2.5 Patterns of regression

Slides were visualized and annotated by two independent researchers (C.G.M & S.K.O.) and unclear slides were resolved by consensus with an expert gastrointestinal pathologist (I.D.N.). Each sample was scored blindly following an externally validated classification diagram that we developed for patterns of tumor response in esophageal cancer (32) and rectal cancer (unpublished data). Tumor patterns of regression were divided into fragmentation (disintegration of the tumor mass in different sized and shaped fragments) or shrinkage (the tumor mass downsizes) (**Supplementary Figure 1A**). The pattern of response was assessed blindly for all patients, regardless if they had had neoadjuvant therapy or not.

### 2.6 Statistical analysis

Statistical analysis was carried out using RStudio version 3.6.2 (Boston, MA, USA, RRID: SCR_000432). One-way ANOVA was done for comparisons between more than two groups in a parametric setting, while Kruskal Wallis one-way ANOVA was used in the non-parametric setting. Student’s T-test was done for comparisons between two groups. Correlations between non-parametric variables were analyzed using Spearman´s rank order correlation test. When comparing categorical variables, a chi-square test was used. A principal component analysis was conducted using singular value decomposition. A P-value lower than 0.05 was considered statistically significant.

## 3 Results

### 3.1 Patient characteristics and treatment schedules

A total of 80 RC patients were included; despite matching, selection bias based on treatment indications remained between the groups (**Table 1**). Gender, differentiation grade, angioinvasion, pN, and pM stage were similar across therapy groups. However, median patient age, tumor location, regression to therapy, lymph node involvement, and pathological T stage were significantly different. Treatment schedule specifics can be found in **Figure 1A**.

### 3.2 Tumor response

An excellent interobserver agreement was reached (κ=0.84). Upon histological evaluation, 14 patients were categorized as “non-responders”, as there was extensive tumor present and no evidence of regression such as fibrosis or mucin. Prevalence of shrinkage and fragmentation was significantly different across therapy groups (**Table 1**, p=0.02, **Supplementary Figure 1B**). Shrinkage was not present in any of the 16 patients treated with CT while being present in 6/16 patients, treated with RCT (**Supplementary Figure 1B**). When analyzing the immune spatial contexture of these patterns of response we did not observe any single or combinations of immune cells that could explain these patterns. However, we observed a tendency towards higher stromal T-cyt, T-reg, and T-helper cells in patients exhibiting a shrinkage pattern of response compared to those with a fragmented pattern (**Supplementary Figure 2**).

### 3.3 Tumor characteristics

Targeted sequencing for microsatellite instable (MSI) markers and 47 cancer-related genes identified pathogenic mutations in 61 of the 64 patients (88%) and MSI in 3 of the 69 patients (4%). The percentages of TP53, KRAS, PIK3CA, and NRAS mutated cases were respectively 74%, 56%, 11%, and 7%. These percentages are in the range of other cohorts (37). The samples with mutations were equally distributed over the different therapies. We then analyzed the relation between the molecular and the immune phenotype, we observed that MSI patients have higher immune cell densities compared to MSS, especially significant are T-cytotoxic cells (p<0.0005), and B cells (p<0.0005)(**Supplementary Figures 3A, 3B**). TP53-mutated tumors have lower immune cell densities compared to TP53-wild type tumors. The most affected immune cells seem to be T-helper cells (p<0.005) and dendritic cells (p<0.005) (**Supplementary Figures 3C, 3D**, respectively). Patients with PIK3CA-mutated tumors had higher tumoral dendritic cell infiltration compared to wild-type tumors (p<0.05, **Supplementary Figure 3E**).

### 3.4 Treatment analysis

**Figure 2:**
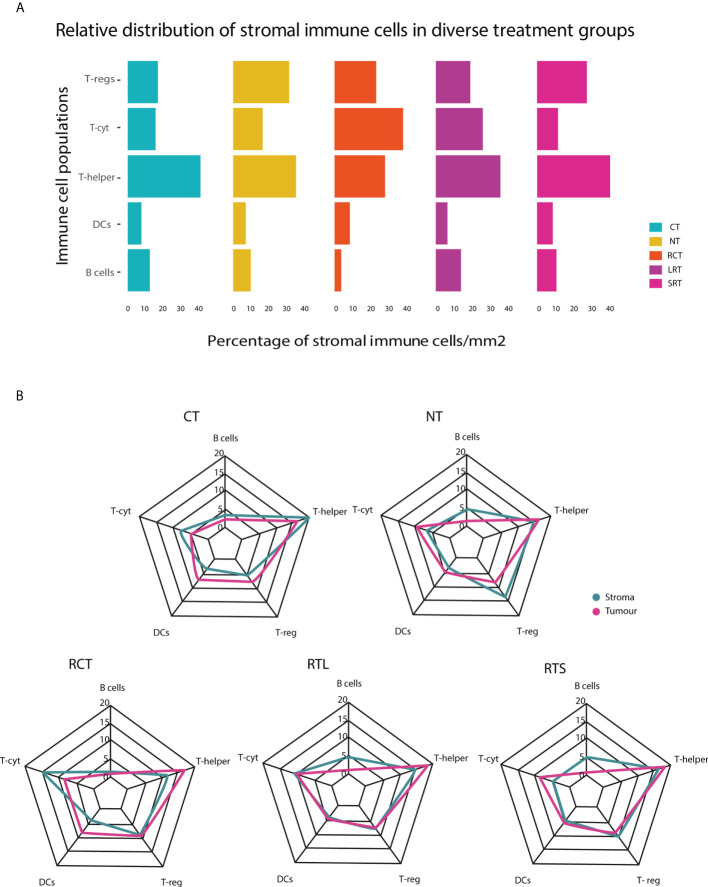
Relative immune cell population in tumor and stroma in diverse treatment settings. **(A)** This stacked bar graph represents the relative distribution of immune cells in the stroma surrounding the tumor. **(B)** Relative distribution of immune cells in the tumor and stroma regions for different treatment settings. NT, No therapy; CT, Chemotherapy; RCT, Radiochemotherapy; LRT, Radiotherapy long course; SRT, Radiotherapy short course.

The relative distribution of stromal immune cells showed T-helper cells as the predominant immune cell populations across therapies (**Figure 2A**). Moreover, a significantly higher density of absolute distribution of immune cells was observed in the stroma compared to the tumor infiltration (**Supplementary Figure 4A**). Immune infiltration in the tumor region did not show significant differences among therapies (**Supplementary Figures 4B-F**).

Differences in the immunophenotype per treatment could be observed (**Figure 2B**, **Supplementary Figure 4A**). T-helper cells were the most predominant population in the stroma of CT, NT, RTL, and RTS treated patients. RCT-treated patients had lower stromal T-helper cells and, had a higher population of T-cyt cells compared to all other treatments. Furthermore, differences could be observed between the stroma and the tumor region, as DCs were one of the predominant types in the tumor region after T-helper cells, something that was not observed in the stromal region.

**Figure 3:**
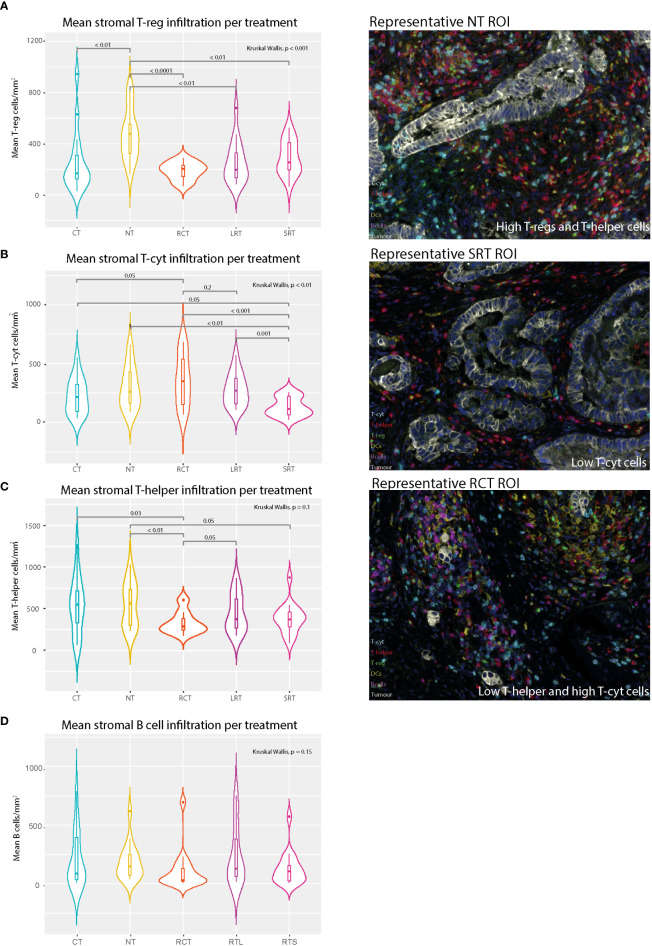
Mean stromal immune cell populations and representative immunofluorescence multiplex images. **(A)** Mean stromal T-reg density in different treatment settings. **(B)** Mean Stromal T-cytotoxic cell density in different treatment settings. **(C)** Mean stromal T-helper density per treatment regimen. **(D)** Mean stromal B cells. NT, No therapy; CT, Chemotherapy; RCT, Radiochemotherapy; LRT, Radiotherapy long course; SRT, Radiotherapy short course.

The stromal T-reg population was less present after any form of neoadjuvant therapy compared to NT, regardless of the waiting time (LRT vs. SRT) (**Figure 3A**, p<0.001). Acute radiotherapy effects were evaluated by comparing the SRT-treated group with the LRT-treated group. The presence of stromal T-cyt cells was significantly lower in SRT-treated patients (**Figure 3B**, p<0.01), especially compared to LRT-treated patients (p=0.001). A tendency toward lower T-helper cell density in SRT-treated patients compared to non-treated patients was also found (**Figure 3C**, p=0.05).

The impact of CT was evaluated in two ways, first by comparison with the NT group and secondly, by comparison of the RCT group with the LRT-treated group. The CT-treated group was then compared with the RCT-treated group to determine a potential synergistic effect of CT. Compared to NT, CT showed a decrease in T-regs in the tumor and the stroma, (p=0.05 and p<0.01, respectively **Supplementary Figure 4D** and **Figure 3A**). Differences in the presence of T-regs were observed when comparing LRT with RCT. Additionally, the tumor microenvironment showed higher stromal T-helper cells in LRT compared to RCT treated patients (p=0.045, **Figure 3C**). There was also a trend towards lower stromal B cells (p=0.1, **Figure 3D**) and T-regs (p=0.1, **Figure 3A**) in RCT-treated patients, as well as higher tumoral DCs (p=0.1, **Supplementary Figure 4F**) compared to LRT-treated patients. Moreover, when comparing RCT and CT-treated patients significantly higher stromal T-cyt cells (p=0.05) and significantly lower stromal T-helper cells (p=0.03) were found in RCT-treated patients.

### 3.5 Heterogeneity of the immune response according to therapy

**Figure 4:**
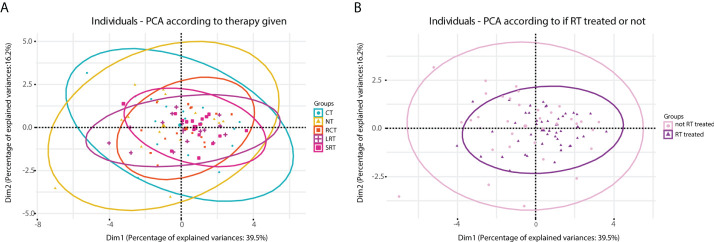
Principal component analysis (PCA) carried out according to therapy given. **(A)**, diagram obtained according to five treatment regimens. **(B)**, diagram obtained when stratified according to treated with RT or no RT. NT, No therapy; CT, Chemotherapy; RCT, Radiochemotherapy; LRT, Radiotherapy long course; SRT, Radiotherapy short course; RT, radiotherapy; noRT, no radiotherapy.

When conducting a principal component analysis (PCA) to reduce the dimensions of the high-plex data, different-sized ellipses of the different treatment groups could be observed (**Figures 4A, B**). Local treatment including RT led to a more homogeneous overall immune response (smaller ellipse) compared to that of patients receiving no treatment or CT (large ellipse) only. Combining dimensions one and two we can explain around 56% of the variance. By analyzing the contribution of each variable to this overall variance we observed that the key players were T-helper cells.

### 3.6 Interaction between the network of immune cells

**Figure 5:**
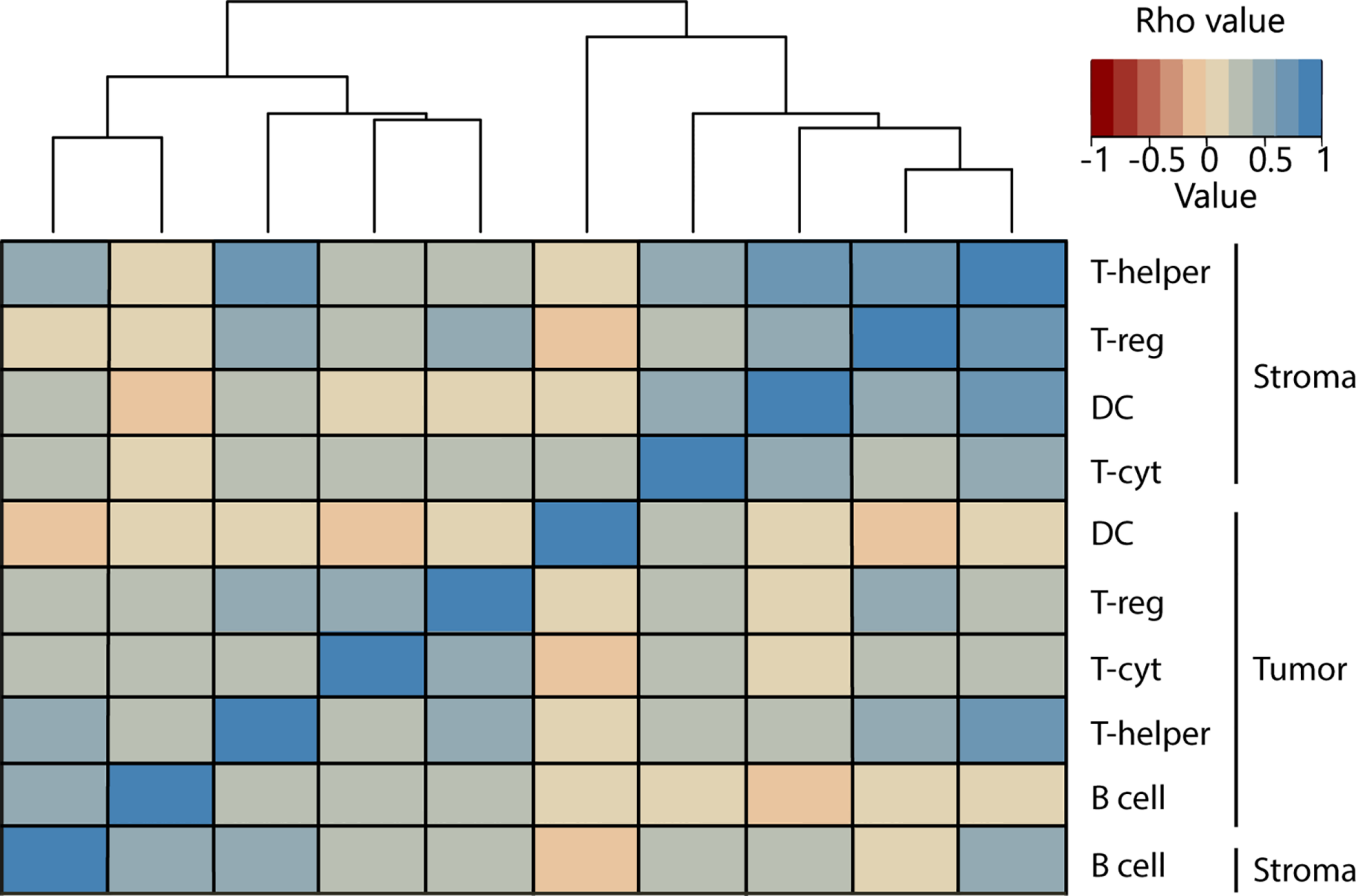
Correlation matrix explains the interaction between the network of immune cells.

By grouping all treatment groups together we were able to analyze the overall immune spatial contexture and interplay between immune populations. There was a consistent correlation between the presence of immune cell subsets in different regions, where immune cell populations were positively correlated in stroma and tumor (**Figure 5**). Moderate correlations were present between the stromal populations of DCs, T-helper, T-reg cells, and T-cyt cells, with the strongest correlation being between stromal T-helper cells and T-regs (rs=0.65). Weaker correlations were observed in the tumor infiltrate, where the main correlations were found between T-regs, T-helper, and T-cyt cells. For more complex interactions, we demonstrated that the presence of T-helper cells positively correlated with all other immune cell populations in all compartments except for tumor-infiltrating DCs, suggesting that these cells behave differently from the rest of immune cell types, with, in many cases, weak negative correlations (**Figure 5**).

### 3.7 Exploratory analysis

#### 3.7.1 Sub-analysis including brachytherapy treatment

A small exploratory cohort of five patients treated with brachytherapy (endoluminal radiotherapy) was also studied to investigate the immunophenotype as a result of this intense local form of therapy. Since three out of the five patients achieved a complete pathological response, we were only able to compare the tumor microenvironment in these five patients to those of the differently-treated patients, as a sample size of two for the tumor region would be biased and, therefore, not a reliable comparison. Strikingly, stromal T-helper, T-cyt, and T-regs showed very low immune densities compared to patients with other treatment regimens (**Supplementary Figures 5A-C**).

## 4 Discussion

Many studies have attempted to study the tumor microenvironment in RC (4, 10, 21, 24, 30, 38–41). One study (17) even compared the tumor immune microenvironment between RC and CRC, where RC patients seem to have lower levels of immune activation. To the best of our knowledge, this is the first attempt to discover the particularities of the immune contexture in relation to treatment strategy and subsequent tumor response. By comparing different treatment groups, we are able to contribute specific effects to treatment modalities and timing. We have shown that T-regs are less present after any form of therapy, regardless of the treatment interval, suggesting a long and maybe permanent effect on the tumor microenvironment.

Furthermore, we have shown that acute RT (SRT) affects the presence of T-cyt and T-helper cell populations the most. Strikingly, this is less evident with LRT, suggesting repopulation takes place in the interval that distinguishes SRT and LRT, which is in line with previous models (4, 24, 30). Previously, Mezheyeuski (17) has shown that memory T-helper, memory T-cyt cells, and macrophage counts decreased immediately after radiotherapy and were increased after longer treatment intervals. Following this idea, the sub-analysis including BT-treated patients evidenced that a higher dose of radiation depletes the T-cyt and T-helper cell populations further than other therapies. This is especially striking given that the waiting period from end of therapy to surgery is equal to that of LRT, so either this interval is not enough to repopulate the tumor microenvironment after such an intense dose, or the mechanisms that control the repopulation process have been damaged.

Moreover, considerably fewer T-helper and T-regs were found in the RT-treated group when it was compared to non-RT-treated (**Supplementary Figures 5D, E**). This occurrence had been previously reported (42) and stated that T cell levels begin to decrease after RT is given and do not reach unirradiated levels until approximately two weeks after completion of treatment. Perhaps due to this somewhat coordinated response after the refractory period following RT, the immune response in RT-treated patients seems to be more homogeneous compared to that of non-RT-treated patients (as can be observed in **Figure 4**). Thus, taking into account these results and given that RT is a more localized treatment compared to CT, we hypothesize that the local microenvironment is more affected by RT and less affected by CT.

Finally, the synergistic effect of radiotherapy in combination with chemotherapy seems to increase the presence of T-cyt cells in the stroma and to decrease the presence of T-helper cells when compared to CT-only treated patients. Furthermore, differences in several immune cell populations were found when comparing LRT and RCT, despite the similar interval and RT regimen. Tumor-infiltrating DCs were found to be higher in RCT-treated patients compared to LRT-treated patients. RCT-treated patients also showed lower stromal T-helper, B cells, and T-regs compared to LRT-treated patients. This suggests that the synergistic effect of combination therapy induces proliferation of T-cyt cells but depletion of other immune cells in the stroma and induces DC tumor infiltration compared to LRT-treated patients.

To study spatial neighborhood relationships, we used multiplex immunohistochemistry, which allowed us to look at cell population interactions. High stromal densities result in more immune cells in the vicinity of the primary tumor, allowing infiltration. In addition, the study of multiple types of cells allows for the analysis of complex interactions. Indeed, as expected (43, 44), the presence of T-helper cells was associated with all other immune cell populations (**Figure 5**). Recent studies have found many correlations between specific immune cell types in different regions (tumor and/or stroma) and prognosis in RC patients (21, 22). When we examined the interregional interactions, we confirmed the general premise that higher stromal cells correlate with higher immune infiltrate and we also found correlations between stromal DCs and T-cyt cells (**Figure 5**). It seems plausible that the reason for this correlation could be cross-priming, where DCs present in the tumor microenvironment activate T-cyt cells by cross-presenting antigens, generating anti-tumor immunity. Further research to confirm this is needed.

Further, we analyzed the spatial contexture of response in a broader sense, linking the histological classification of response with the immune microenvironment. We found that patterns of response could not be explained by a single or combination of multiple immune cell populations across different therapies. Nevertheless, a tendency towards higher stromal T-cyt, T-reg, and T-helper cells in patients exhibiting a shrinkage pattern of response compared to those with a fragmented pattern (**Supplementary Figure 2**) was observed. A plausible explanation could be that the coordinated presence of several types of immune cell lineages is necessary for a more effective cancer cell elimination, which seems to be the shrinkage pattern of response compared to the fragmented pattern.

Since our study included a heterogeneous group of patients (due to the different therapies given), outcome was not the main aim of our study and therefore we could not analyze immune populations for prognostic value. Nevertheless, a few studies had described a correlation between T-regs and improved survival (23, 24), which we could also observe in our cohort, regardless of treatment (data not shown).

There were several limitations to this study. Firstly, it was a retrospective study with relatively small patient groups from one single institution. Since RCT is the standard therapy for locally advanced RC, we could not expand the other treatment groups without introducing significant selection bias for patient and tumor characteristics. No outcome data could be reported since there was no randomization between the different treatment arms. Similarly, outcomes such as downstaging could not be compared since the different treatment groups did not have the same interval between therapy and surgery. The objective of this study was to explore the spatial and immune contexture after neoadjuvant therapy in rectal cancer to provide insight into their interplay as we move towards local and tailored treatments. Our goal was to unveil the unique immune infiltrate of the tumor and tumor microenvironment of differentially treated rectal cancer patients.

Further research should focus on unraveling the link between different patterns of response and the immune cell infiltrate. Potential therapeutic applications may arise as stimulating certain immune cell lineages could influence the pattern of response to treatment, which can be used to favor a shrinkage pattern environment instead of a fragmented or no response pattern. Moreover, the prognostic relevance of T-regs needs to be investigated in different regions of the primary tumor as its role in the stromal and tumor regions could be different.

In conclusion, we believe that this study is the first to report the differential effects of specific neoadjuvant therapies on the immune contexture of advanced rectal cancer. We have shown that many immune populations (including T-helper, T-cyt, and T-reg cells) are affected mainly by radiotherapy treatment. The re-emergence (or lack) of specific immune cell populations after treatment over time might be linked to tumor regression. Therefore, a better understanding of the reorganization of the immune contexture after therapy is important for the appropriate management of locally advanced RC patients.

## Data availability statement

The original contributions presented in the study are included in the article/**Supplementary Material**. Further inquiries can be directed to the corresponding authors.

## Ethics statement

Ethical review and approval was not required for the study on human participants in accordance with the local legislation and institutional requirements. Written informed consent for participation was not required for this study in accordance with the national legislation and the institutional requirements.

## Author contributions

The following detailed Credit author statement is added by the corresponding author, who is responsible for ensuring that the descriptions are accurate and agreed by all authors:

CG: methodology, formal analysis, data curation, writing - original draft preparation, visualization, and writing - review and editing. YB: methodology, formal analysis, data curation, and writing - original draft preparation. SKÖ: methodology, validation, and writing - review and editing. MA: interpretation of data, original draft preparation, and writing - review and editing. MG: methodology, data collection, interpretation of data, and writing - review and editing. SV: methodology and writing - review and editing. CM: conceptualization, data curation, writing - review and editing, and supervision. IN: conceptualization, methodology, validation, data curation, writing - review and editing, and supervision. All authors contributed to the article and approved the submitted version.

## Funding

This work received funding from the Dutch Cancer Society in two grants; Alpe d’HuZes/KWF program grant (KWF UL 2013-6311) and a KWF grant (10602/2016-2).

## Conflict of interest

The authors declare that the research was conducted in the absence of any commercial or financial relationships that could be construed as a potential conflict of interest.

## Publisher’s note

All claims expressed in this article are solely those of the authors and do not necessarily represent those of their affiliated organizations, or those of the publisher, the editors and the reviewers. Any product that may be evaluated in this article, or claim that may be made by its manufacturer, is not guaranteed or endorsed by the publisher.

## References

[1] Brouwer NPM Bos A Lemmens V Tanis P.J Hugen N Nagtegaal ID. An overview of 25 years of incidence, treatment and outcome of colorectal cancer patients. Int J Cancer (2018) 143(11):2758–66. doi: 10.1002/ijc.31785 PMC6282554 30095162

[2] Gijn van W Marijnen CAM Nagtegaal ID Kranenbarg EM-K Putter H Wiggers T. Preoperative radiotherapy combined with total mesorectal excision for resectable rectal cancer: 12-year follow-up of the multicentre, randomised controlled TME trial. Lancet Oncol (2011) 12(6):575–82. doi: 10.1016/S1470-2045(11)70097-3 21596621

[3] Kapiteijn E Marijnen CA Nagtegaal ID Putter H Steup WH Wiggers T. Preoperative radiotherapy combined with total mesorectal excision for resectable rectal cancer. N Engl J Med (2001) 345(9):638–46. doi: 10.1056/NEJMoa010580 11547717

[4] Glynne-Jones R Hall M Nagtegaal ID. The optimal timing for the interval to surgery after short course preoperative radiotherapy (5 x5 gy) in rectal cancer - are we too eager for surgery? Cancer Treat Rev (2020) 90:102104. doi: 10.1016/j.ctrv.2020.102104 33002819

[5] Fischer J Eglinton TW Richards SJ Frizelle FA. Predicting pathological response to chemoradiotherapy for rectal cancer: a systematic review. Expert Rev Anticancer Ther (2021) 21(5):489–500. doi: 10.1080/14737140.2021.1868992 33356679

[6] Bahadoer RR Dijkstra EA Etten van B Marijnen CAM Putter H Kranenbarg EM-K. Short-course radiotherapy followed by chemotherapy before total mesorectal excision (TME) versus preoperative chemoradiotherapy, TME, and optional adjuvant chemotherapy in locally advanced rectal cancer (RAPIDO): a randomised, open-label, phase 3 trial. Lancet Oncol (2021) 22(1):29–42. doi: 10.1016/S1470-2045(20)30555-6 33301740

[7] Erlandsson J Lorinc E Ahlberg M Pettersson D Holm T Glimelius B. Tumour regression after radiotherapy for rectal cancer - results from the randomised Stockholm III trial. Radiother Oncol (2019) 135:178–86. doi: 10.1016/j.radonc.2019.03.016 31015165

[8] Rombouts AJM Hugen N Verhoeven RHA Elferink MAG Poortmans PMP Nagtegaal ID. Tumor response after long interval comparing 5x5Gy radiation therapy with chemoradiation therapy in rectal cancer patients. Eur J Surg Oncol (2018) 44(7):1018–24. doi: 10.1016/j.ejso.2018.03.017 29678303

[9] Suwanthanma W Kitudomrat S Euanorasetr C. Clinical outcome of neoadjuvant chemoradiation in rectal cancer treatment. Med (Baltimore) (2021) 100(38):e27366. doi: 10.1097/MD.0000000000027366 PMC8462585 34559161

[10] Park IJ An S Kim SY Lim HM Hong SM Kim MJ. Prediction of radio-responsiveness with immune-profiling in patients with rectal cancer. Oncotarget (2017) 8(45):79793–802. doi: 10.18632/oncotarget.19558 PMC5668093 29108360

[11] Nagtegaal ID Marijnen CA Kranenbarg EK Mulder-Stapel A Hermans J Velde van CJ. Local and distant recurrences in rectal cancer patients are predicted by the nonspecific immune response; specific immune response has only a systemic effect–a histopathological and immunohistochemical study. BMC Cancer (2001) 1:7. doi: 10.1186/1471-2407-1-7 11481031 PMC35356

[12] Perez-Ruiz E Berraondo P. Immunological landscape and clinical management of rectal cancer. Front Immunol (2016) 7:61. doi: 10.3389/fimmu.2016.00061 26941741 PMC4761957

[13] Reimers MS Engels CC Putter H Morreau H Liefers GJ Velde van CJ. Prognostic value of HLA class I, HLA-e, HLA-G and tregs in rectal cancer: a retrospective cohort study. BMC Cancer (2014) 14:486. doi: 10.1186/1471-2407-14-486 24997850 PMC4094545

[14] McMullen TP Lai R Dabbagh L Wallace TM Gara CJ. Survival in rectal cancer is predicted by T cell infiltration of tumour-associated lymphoid nodules. Clin Exp Immunol (2010) 161(1):81–8. doi: 10.1111/j.1365-2249.2010.04147.x PMC2940152 20408858

[15] Iseas S Sendoya JM Robbio J Coraglio M Kujaruk M Mikolaitis V. Prognostic impact of an integrative landscape of clinical, immune, and molecular features in non-metastatic rectal cancer. Front Oncol (2021) 11:801880. doi: 10.3389/fonc.2021.801880 35071006 PMC8777220

[16] Zhang L Zhao Y Dai Y Cheng JN Gong Z Feng Y. Immune landscape of colorectal cancer tumor microenvironment from different primary tumor location. Front Immunol (2018) 9:1578. doi: 10.3389/fimmu.2018.01578 30042763 PMC6048410

[17] Mezheyeuski A Micke P Martin-Bernabe A Backman M Hrynchyk I Hammarstrom K. The immune landscape of colorectal cancer. Cancers (Basel) (2021) 13(21). doi: 10.3390/cancers13215545 PMC8583221 34771707

[18] Galon J Pages F Marincola FM Angell H.K Thurin M Lugli A. Cancer classification using the immunoscore: a worldwide task force. J Transl Med (2012) 10:205. doi: 10.1186/1479-5876-10-205 23034130 PMC3554496

[19] Nagorsen D Voigt S Berg E Stein H Thiel E Loddenkemper C. Tumor-infiltrating macrophages and dendritic cells in human colorectal cancer: relation to local regulatory T cells, systemic T-cell response against tumor-associated antigens and survival. J Transl Med (2007) 5:62. doi: 10.1186/1479-5876-5-62 18047662 PMC2212626

[20] Janco Tran JM Lamichhane P Karyampudi L Knutson KL. Tumor-infiltrating dendritic cells in cancer pathogenesis. J Immunol (2015) 194(7):2985–91. doi: 10.4049/jimmunol.1403134 PMC4369768 25795789

[21] Edin S Kaprio T Hagstrom J Larsson P Mustonen H Bockelman C. The prognostic importance of CD20(+) b lymphocytes in colorectal cancer and the relation to other immune cell subsets. Sci Rep (2019) 9(1):19997. doi: 10.1038/s41598-019-56441-8 PMC6934737 31882709

[22] Mlecnik B Eynde den Van M Bindea G Church SE Vasaturo A Fredriksen T. Comprehensive intrametastatic immune quantification and major impact of immunoscore on survival. J Natl Cancer Inst (2018) 110(1):97–108. doi: 10.1093/jnci/djx123 28922789

[23] Kuwahara T Hazama S Suzuki N Yoshida S Tomochika S Nakagami Y. Intratumoural-infiltrating CD4 + and FOXP3 + T cells as strong positive predictive markers for the prognosis of resectable colorectal cancer. Br J Cancer (2019) 121(8):659–65. doi: 10.1038/s41416-019-0559-6 PMC6889292 31488881

[24] Yasui K Kondou R Iizuka A Miyata H Tanaka E Ashizawa T. Effect of preoperative chemoradiotherapy on the immunological status of rectal cancer patients. J Radiat Res (2020) 61(5):766–75. doi: 10.1093/jrr/rraa041 PMC7482156 32672335

[25] Schnellhardt S Hirneth J Buttner-Herold M Daniel C Haderlein M Hartmann A. The prognostic value of FoxP3+ tumour-infiltrating lymphocytes in rectal cancer depends on immune phenotypes defined by CD8+ cytotoxic T cell density. Front Immunol (2022) 13:781222. doi: 10.3389/fimmu.2022.781222 PMC8818710 35140715

[26] Georges NDF Oberli B Rau TT Galvan JA Nagtegaal ID Dawson H. Tumour budding and CD8(+) T cells: 'attackers' and 'defenders' in rectal cancer with and without neoadjuvant chemoradiotherapy. Histopathology (2021) 78(7):1009–18. doi: 10.1111/his.14319 33340423

[27] Yasuda K Nirei T Sunami E Nagawa H Kitayama J. Density of CD4(+) and CD8(+) T lymphocytes in biopsy samples can be a predictor of pathological response to chemoradiotherapy (CRT) for rectal cancer. Radiat Oncol (2011) 6(1):49–55. doi: 10.1186/1748-717X-6-49 PMC3120676 21575175

[28] Pages F Berger A Camus M Sanchez-Cabo F Costes A Molidor R. Effector memory T cells, early metastasis, and survival in colorectal cancer. N Engl J Med (2005) 353(25):2654–66. doi: 10.1056/NEJMoa051424 16371631

[29] Szynglarewicz B MR Suder E Sydor D Forgacz J Pudełko M Grzebieniak Z. Predictive value of lymphocytic infiltration and character of invasive margin following total mesorectal excision with sphincter preservation for the high-risk carcinoma of the rectum. Adv Med Sci (2007) 52:159–63. 18217410

[30] Lee YJ Lee SB Beak SK Han YD Cho MS Hur H. Temporal changes in immune cell composition and cytokines in response to chemoradiation in rectal cancer. Sci Rep (2018) 8(1):7565. doi: 10.1038/s41598-018-25970-z PMC5953940 29765096

[31] Wu Z Zhang J Cai Y Deng R Yang L Li J. Reduction of circulating lymphocyte count is a predictor of good tumor response after neoadjuvant treatment for rectal cancer. Med (Baltimore) (2018) 97(38):e11435. doi: 10.1097/MD.0000000000011435 PMC6160071 30235653

[32] Martinez Graham C Ozturk Kus S Al-Kaabi A Valkema MJ Bokhorst JM Rosman C. Shrinkage versus fragmentation response in neoadjuvantly treated oesophageal adenocarcinoma: significant prognostic relevance. Histopathology (2022) 80(6):982–94. doi: 10.1111/his.14644 PMC9325353 35352847

[33] Akoya Biosciences I. Opal multiplex IHC assay development guide and image acquisition information phenoptics research solutions. Akoya Biosciences, Inc. (2019) Available at: https://www.akoyabio.com/phenoptics/opal-kits-reagents/opal-7-tumor-infiltrating-lymphocyte-kit/.

[34] Gorris MAJ Halilovic A Rabold K Duffelen van A Wickramasinghe IN Verweij D. Eight-color multiplex immunohistochemistry for simultaneous detection of multiple immune checkpoint molecules within the tumor microenvironment. J Immunol (2018) 200(1):347–54. doi: 10.4049/jimmunol.1701262 29141863

[35] Gorris MAJ Woude der van LL Kroeze LI Bol K Verrijp K Amir AL. Paired primary and metastatic lesions of patients with ipilimumab-treated melanoma: high variation in lymphocyte infiltration and HLA-ABC expression whereas tumor mutational load is similar and correlates with clinical outcome. J Immunother Cancer (2022) 10(5). doi: 10.1136/jitc-2021-004329 PMC9109111 35550553

[36] Sultan S Gorris MAJ Woude der van LL Buytenhuijs F Martynova E Wilpe van S. A segmentation-free machine learning architecture for immune land-scape phenotyping in solid tumors by multichannel imaging. bioRxiv (2021), 2021. doi: 10.1101/2021.10.22.464548

[37] Cancer Genome Atlas N. Comprehensive molecular characterization of human colon and rectal cancer. Nature (2012) 487(7407):330–7. doi: 10.1038/nature11252 PMC3401966 22810696

[38] Shinto E Hase K Hashiguchi Y Sekizawa A Ueno H Shikina A. CD8+ and FOXP3+ tumor-infiltrating T cells before and after chemoradiotherapy for rectal cancer. Ann Surg Oncol (2014) . 21 Suppl 3:S414–21. doi: 10.1245/s10434-014-3584-y 24566864

[39] Anitei MG Zeitoun G Mlecnik B Marliot F Haicheur N Todosi AM. Prognostic and predictive values of the immunoscore in patients with rectal cancer. Clin Cancer Res (2014) 20(7):1891–9. doi: 10.1158/1078-0432.CCR-13-2830 24691640

[40] Angell HK Bruni D Barrett JC Herbst R Galon J. The immunoscore: Colon cancer and beyond. Clin Cancer Res (2020) 26(2):332–9. doi: 10.1158/1078-0432.CCR-18-1851 31413009

[41] Mlecnik B Bifulco C Bindea G Marliot F Lugli A Lee JJ. Multicenter international society for immunotherapy of cancer study of the consensus immunoscore for the prediction of survival and response to chemotherapy in stage III colon cancer. J Clin Oncol (2020), JCO1903205. doi: 10.1200/JCO.19.03205 PMC7605397 32897827

[42] Nagtegaal ID Marijnen CA Kranenbarg EK Mulder-Stapel A Hermans J Velde van CJ. Short-term preoperative radiotherapy interferes with the determination of pathological parameters in rectal cancer. J Pathol (2002) 197(1):20–7. doi: 10.1002/path.1098 12081199

[43] Alberts B JA Lewis J. T Cells and MHC proteins. In: Molecular biology of the cell, g. science. New York: Science Garland (2002).

[44] Bennett SR Carbone FR Karamalis F Miller JF Heath WR. Induction of a CD8+ cytotoxic T lymphocyte response by cross-priming requires cognate CD4+ T cell help. J Exp Med (1997) 186(1):65–70. doi: 10.1084/jem.186.1.65 PMC2198964 9206998

