# Supplementary material for: The immune microenvironment landscape shows treatment-specific differences in rectal cancer patients
Source: Front Immunol. 2022 Sep 27;13:1011498. doi: 10.3389/fimmu.2022.1011498 (PMC9552175; doi:10.3389/fimmu.2022.1011498)
Supplement: Supplementary file 1 [file DataSheet_1.docx]

Supplementary Material

# Supplementary Figures and Tables

## Supplementary Figures


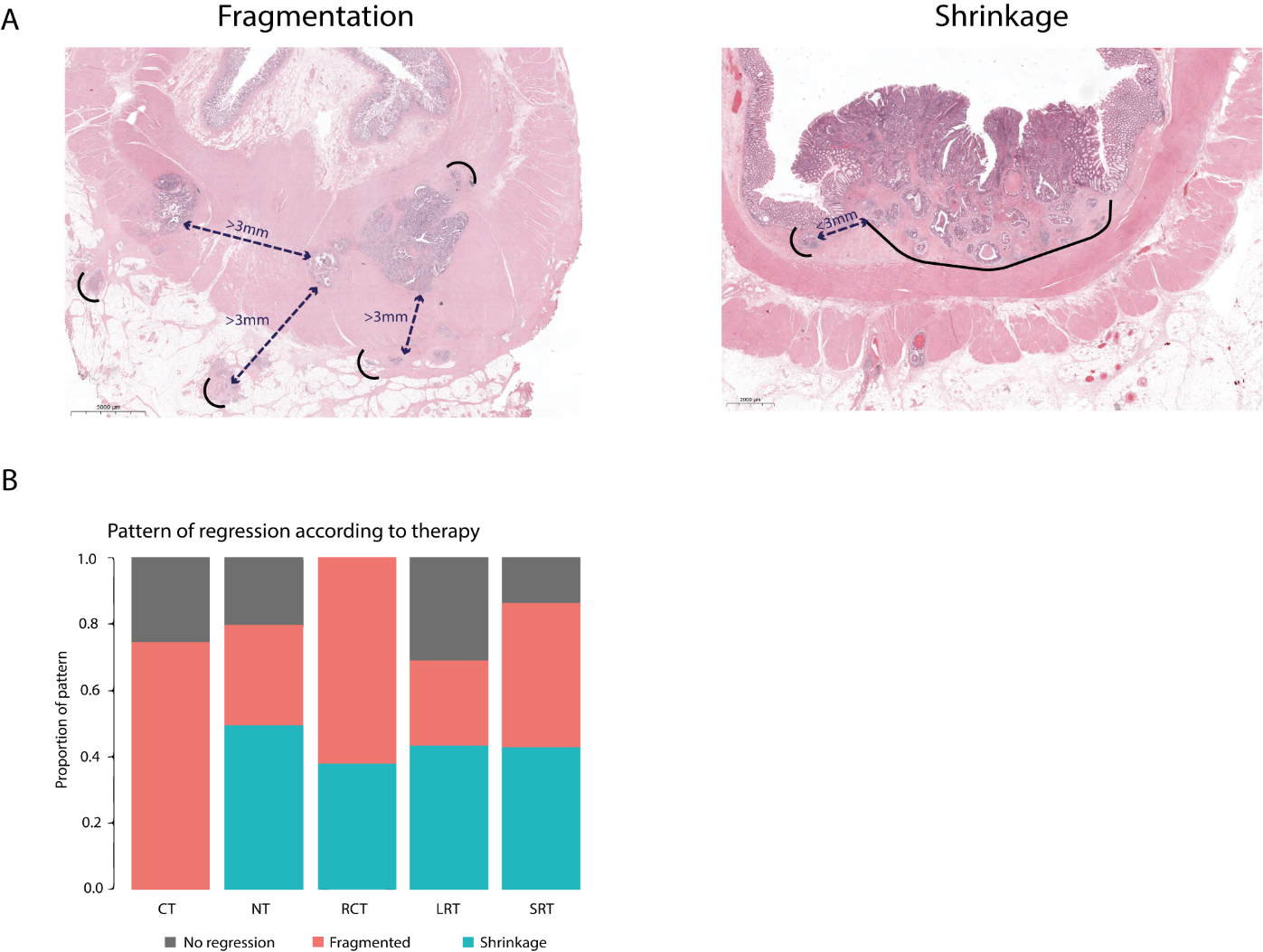


**Supplementary Figure 1.** Tumor patterns of response after neoadjuvant therapy. A. Tumor patterns of regression were divided into fragmentation (disintegration of the tumor mass in differently sized and shaped fragments further away than 3mm from the tumor border) or shrinkage (the tumor mass remains a bulk that can contain fragments within 3mm distance from the border). B. prevalence of Shrinkage and Fragmentation was different across therapy groups. Fragmentation was present in 12/16 patients treated with CT and in 10/16 patients treated with RCT, significantly different than the other two groups (4/16 for LRT and 7/16 for SRT). NT is included because cases were scored blindly but these cases were not included in the estimation of prognostic value. C. Prognostic value of the patterns of response regardless of therapy showing a survival benefit of shrinkage over fragmentation. NT= No therapy, CT= Chemotherapy, RCT = Radiochemotherapy, LRT = Radiotherapy long course, SRT= Radiotherapy short course.


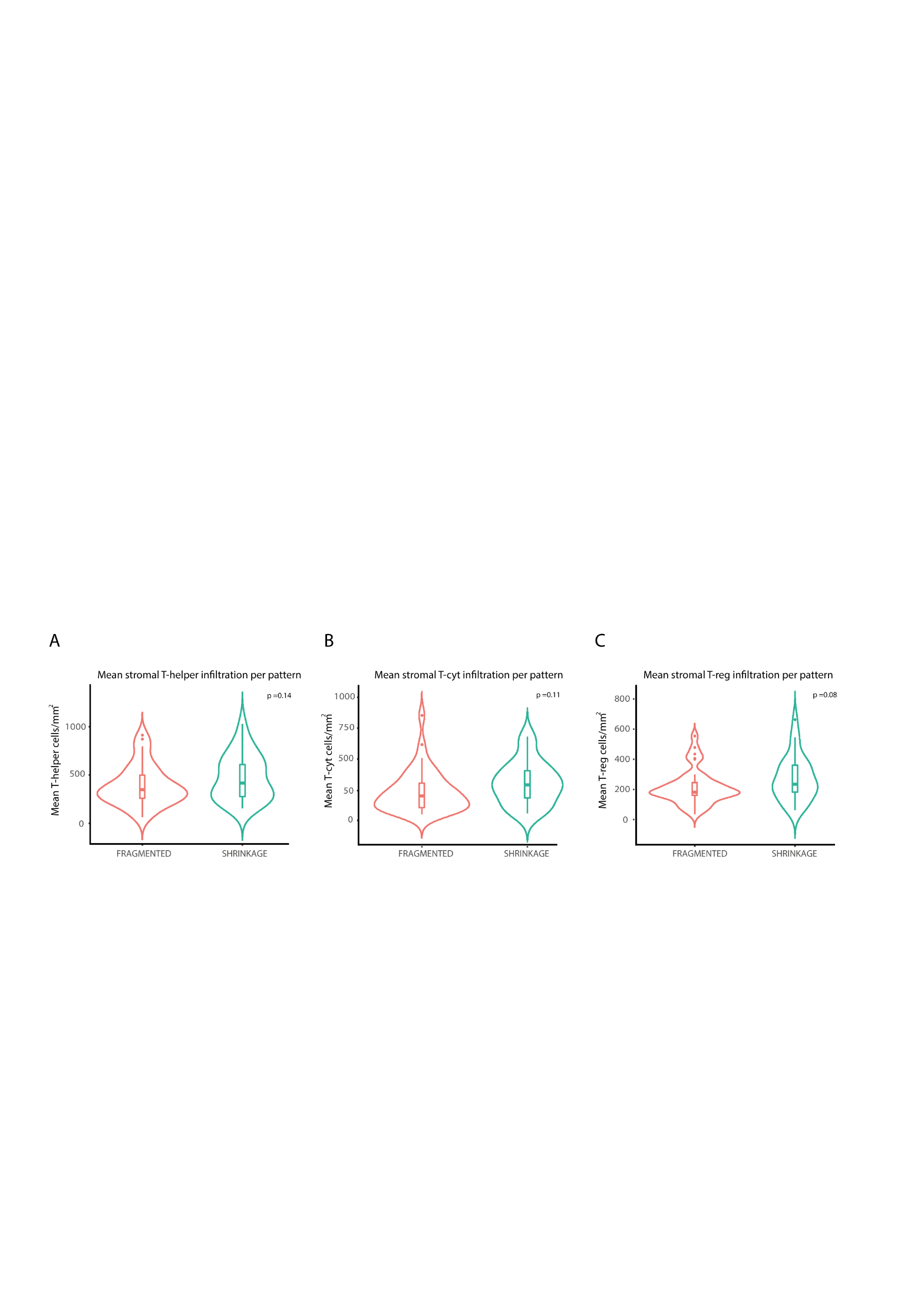


**Supplementary Figure 2**. Immune stromal infiltration according to pattern of response. A, Stromal T-helper cell density. B, Stromal T-cyt cell density. C. Stromal T-reg density. In all cases there is a tendency towards higher stromal T-cyt, T-reg and T-helper cells in patients exhibiting a shrinkage pattern of response compared to those with a fragmented pattern.


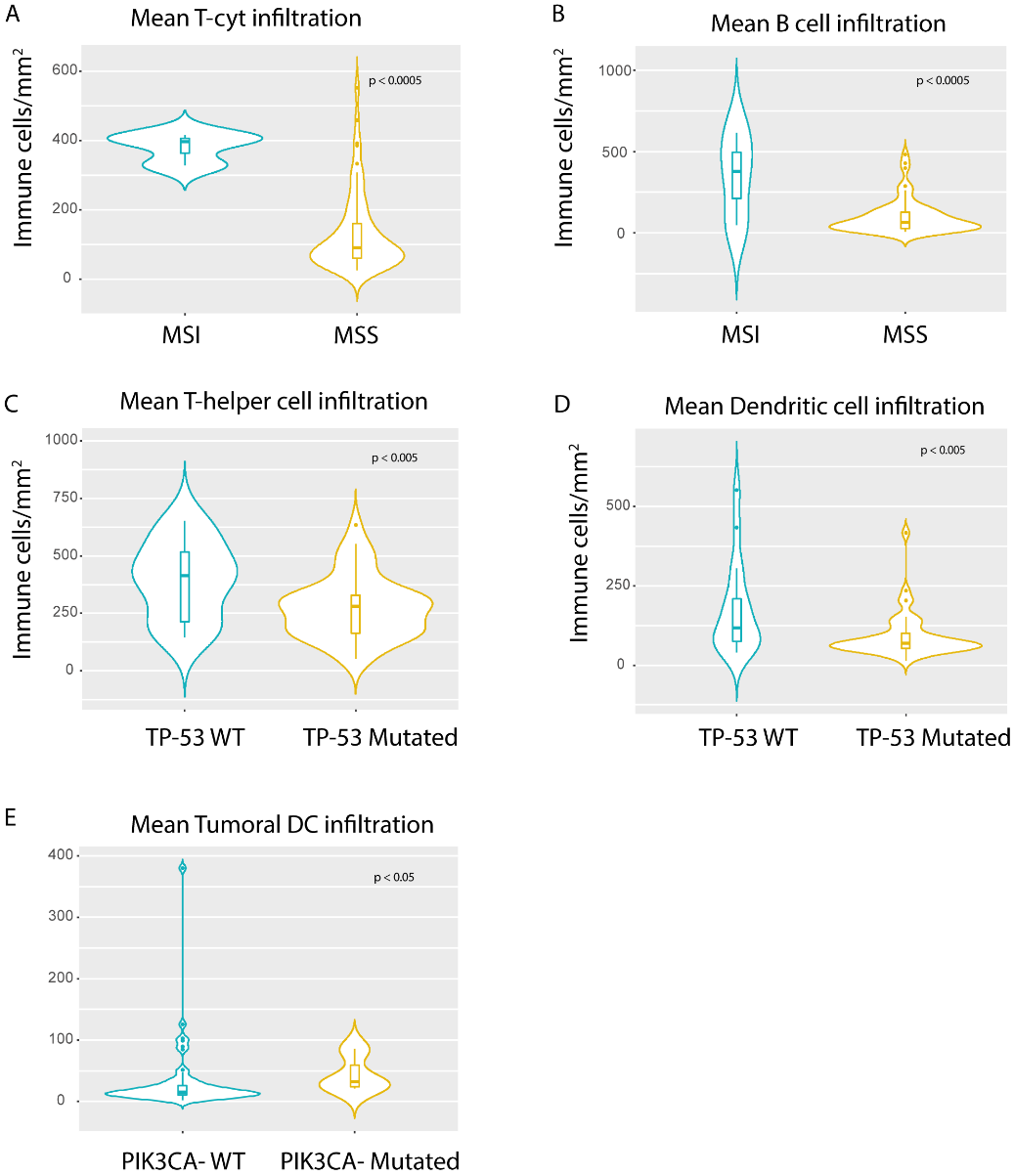


**Supplementary Figure 3**. Relation between the molecular and the immune phenotype. A-B Higher overall T-cyt and B cell infiltration was found in MSI patients compared to MSS patients. C-D, TP53-mutated tumors have lower T-helper cells and DCs compared to TP53-wild type tumors. E, Patients with PIK3CA-mutated tumors had higher tumoral dendritic cell infiltration compared to wild type tumors.


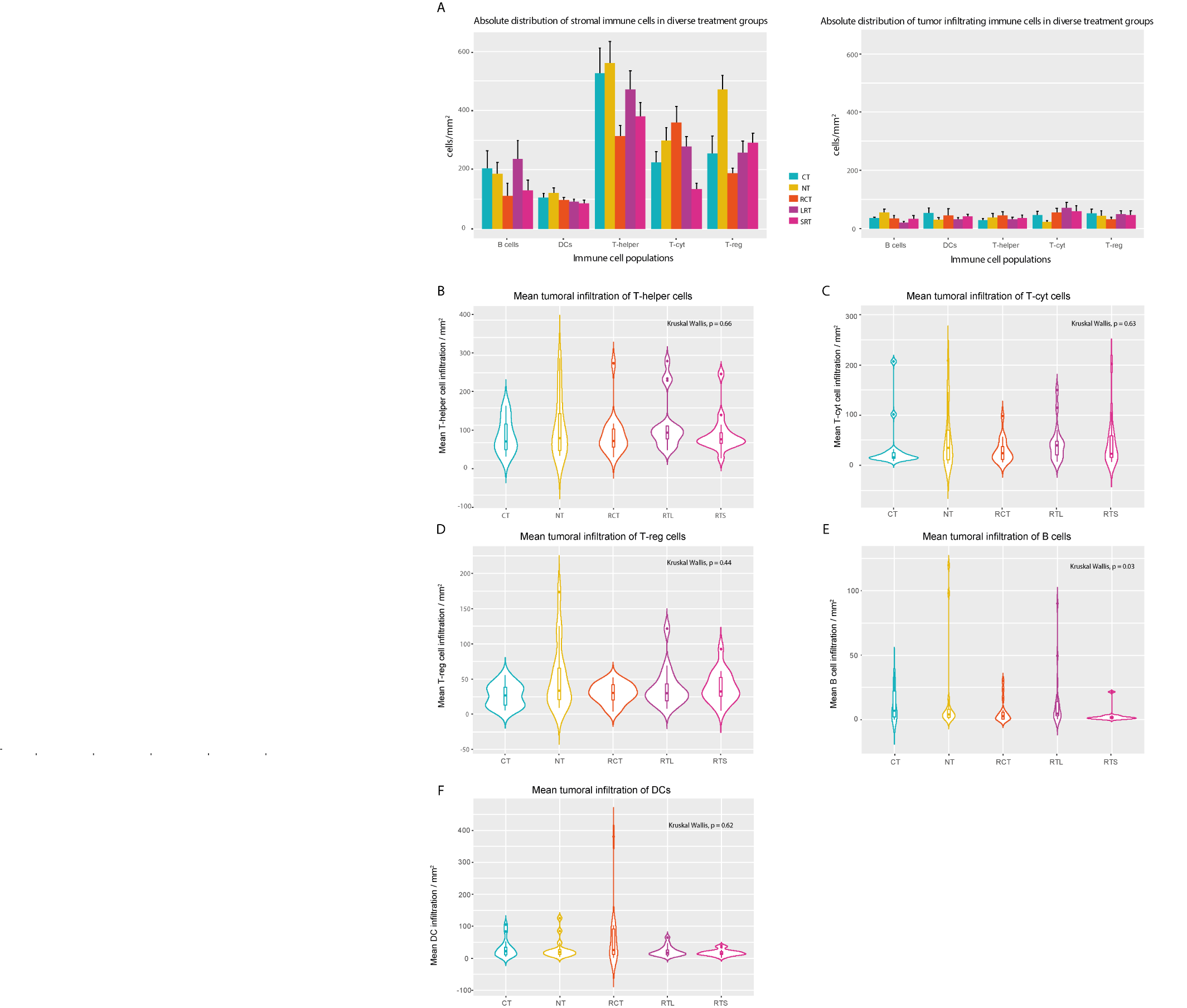


**Supplementary Figure 4.** Absolute distribution of immune cells in stroma and tumor and mean tumor-infiltrating immune cell densities according to therapy. A. Comparison of absolute distribution of immune cells in the stroma and the tumor. As expected, there are more immune cells in the stroma region compared to the tumor. B. Mean tumor-infiltrating T-helper cells, C, T-cyt cells and D, T-reg cells across different therapies show no significant differences. E. Mean tumor-infiltrating B cells shows significant differences among therapies, which need to be taken with caution as these numbers are very small. F. Mean tumor-infiltrating DCs across different therapies show no significant differences. NT= No therapy, CT= Chemotherapy, RCT = Radiochemotherapy, LRT = Radiotherapy long course, SRT= Radiotherapy short course.


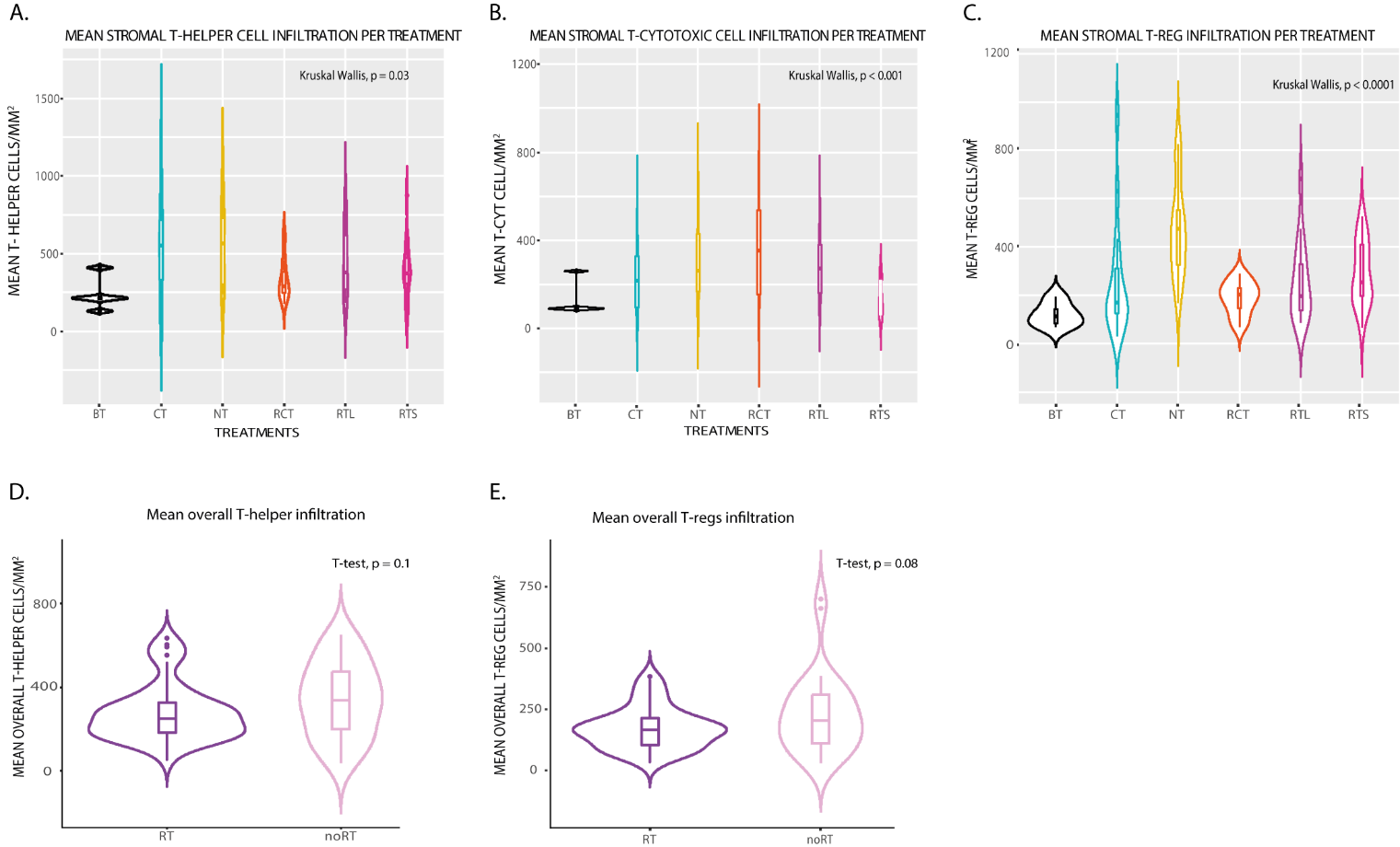


**Supplementary Figure 5**. Sub-analysis including a small cohort of brachytherapy treated patients and the immune cell infiltration when patients are stratified according to general treatment regimens receiving RT or not. A-C stromal T-helper, T-cyt and T-regs (respectively) showed very low immune densities compared to patients with other treatment regimens. D-E Less T-helper and T-regs were found in the RT-treated group when it was compared to non-RT-treated patients. NT= No therapy, CT= Chemotherapy, RCT = Radiochemotherapy, LRT = Radiotherapy long course, SRT= Radiotherapy short course. RT= Radiotherapy

## Supplementary Tables

### Supplementary Table 1. Targeted mutation panel used (PathV3). This was used to determine any mutations that could clearly influence the immune phenotype of patients such as MSI status.

| Gene | RefSeq | Region of interest | Amplification detection | Loss detection | Mutation detection |
| --- | --- | --- | --- | --- | --- |
| AKT1 | NM_005163 | codon 17 |  |  | x |
| AKT2 | NM_001626 | codon 17 |  |  | x |
| AKT3 | NM_181690 | codon 17 |  |  | x |
| ALK | NM_004304 | codon 1059-1278 |  |  | x |
| ARAF | NM_001654 | codon 214 |  |  | x |
| BRAF | NM_004333 | codon 455-488, 566-580, 594-605 | x |  | x |
| B2M | NM_004048 | whole gene |  |  | x |
| CCND1 | NM_053056 | NA | x |  |  |
| CCND3 | NM_001760 | NA | x |  |  |
| CDK4 | NM_000075 | NA | x |  |  |
| CDK6 | NM_001259 | NA | x |  |  |
| CDKN2A (P14/P16) | NM_058195/ NM_000077 | >95% of coding sequences and splice sites (-5/+5) |  | x | x |
| CTNNB1 | NM_001904 | codon 32-45, 333-335, 376-386 |  |  | x |
| EGFR | NM_005228 | codon 434-499, 688-875 | x |  | x |
| ERBB2 | NM_004448 | codon 310, 650-883 | x |  | x |
| ERBB4 | NM_005235 | codon 79-140, 248-399, 431-496, 542-623, 694-734, |  |  | x |
|  |  | 768-829, 908-955, 989-1045, 1162-1309 |  |  |  |
| ESR1 | NM_000125 | codon 380-555 |  |  | x |
| FGFR1 | NM_023110 | codon 158, 283, 546, 577, 592, 656, 687 | x |  | x |
| FGFR2 | NM_000141 | codon 250-392, 522-662 | x |  | x |
| FGFR3 | NM_000142 | codon 248-653 | x |  | x |
| GNA11 | NM_002067 | codon 183, 209 |  |  | x |
| GNAQ | NM_002072 | codon 183, 209 |  |  | x |
| GNAS | NM_000516 | codon 201, 227 |  |  | x |
| HIST1H3B | NM_003537 | codon 27 en 28 |  |  | x |
| HRAS | NM_005343 | codon 12, 13, 27, 59 and 61 |  |  | x |
| H3F3A | NM_002107 | codon 28 and 35 |  |  | x |
| H3F3B | NM_005324 | codon 37 |  |  | x |
| IDH1 | NM_005896 | codon 132 |  |  | x |
| IDH2 | NM_002168 | codon 140 and 172 |  |  | x |
| JAK2 | NM_004972 | codon 617 |  |  | x |
| KEAP1 | NM_203500 | >95% of coding sequences and splice sites (-5/+5) |  |  | x |
| KIT | NM_000222 | codon 412-513, 550-591, 640-787, 799-850 | x |  | x |
| KRAS | NM_004985 | codon 12, 13, 59, 61, 117 and 146 | x |  | x |
| MAP2K1 | NM_002755 | codon 28-231 |  |  | x |
| MDM2 | NM_002392 | NA | x |  |  |
| MET | NM_001127500 | codon 168, 375, 982-1027, 1230-1284 , 1304 | x |  | x |
| MTOR | NM_004958 | codon 1458-1489, 1789-1820, 1971-1995, 2194-2220, |  |  | x |
|  |  | 2404-2433, 2484-2509 |  |  |  |
| MYC | NM_002467 | NA | x |  |  |
| NRAS | NM_002524 | codon 12, 13, 59, 61, 117 and 146 |  |  | x |
| NTRK1 | NM_002529 | codon 342, 545-682 |  |  | x |
| NTRK3 | NM_002530 | codon 623 and 696 |  |  | x |
| PDGFRA | NM_006206 | codon 552-595, 632-667, 824-848 | x |  | x |
| PDGFRB | NM_002609 | codon 528-602, 639-727, 822-862 | x |  | x |
| PIK3CA | NM_006218 | codon 76-118, 345, 420, 539-554, 1043-1050 | x |  | x |
| POLE | NM_006231 | codon 268-491 |  |  | x |
| PTEN | NM_000314 | >95% of coding sequences and splice sites (-5/+5) |  | x | x |
| RAC1 | NM_006908 | codon 29 |  |  | x |
| RAF1 | NM_002880 | codon 257-261 |  |  | x |
| RET | NM_020975 | codon 530, 594-712, 763-933 |  |  | x |

### **Supplementary Table 2**. Optimized Vectra panel of antibodies used in multiplex IHC-IF. The order in the table is the order in which the antibodies were added, as well as their dilutions and corresponding Opal™ colors.

|  | **Antibody** | **Clone** | **Antibody dilution** | **Antigen retrieval** | **Opal™** | **Opal™ dilution** |
| --- | --- | --- | --- | --- | --- | --- |
| 1 | CD8 | C8/144B | 1/200 | EDTA 20min | 690 | 1/50 |
| 2 | CD20 | L26 | 1/600 | EDTA 20min | 570 | 1/50 |
| 3 | CD3 | Sp7 | 1/200 | EDTA 20min | 520 | 1/50 |
| 4 | Foxp3 | 236A/E7 | 1/100 | EDTA 20min | 540 | 1/50 |
| 5 | CD11c | EP1347Y | 1/1000 | EDTA 20min | 620 | 1/50 |
| 6 | AE1/3 | 5D3 | 1/1000 | EDTA 20min | 650 | 1/200 |
